# Oropouche virus disrupts neurodevelopment and is vertically transmitted

**DOI:** 10.21203/rs.3.rs-7512609/v1

**Published:** 2025-09-09

**Authors:** Kellie Jurado, Carl Bannerman, Drake Philip, Taylor Miller-Ensminger, Elizabeth Kennedy, Stephanie Liu, Kristen Walsh, Guo-li Ming

**Affiliations:** University of Pennsylvania; University of Pennsylvania; University of Pennsylvania; University of Pennsylvania; University of Pennsylvania; University of Pennsylvania; University of Pennsylvania; University of Pennsylvania

## Abstract

Oropouche virus (OROV) historically caused a self-limiting disease, yet recent strains are associated with congenital infection and neurodevelopmental disruption. These cases highlight a need to study OROV as a congenital pathogen and determine the impact of infection on neurodevelopment. Here, we show that OROV is vertically transmitted and induces a microcephaly-like phenotype in human forebrain organoids. We found OROV robustly infects human neural progenitor cells in organoids. In contrast to ZIKV, OROV had a heightened capacity for infection and organoid pathology. We show this increased pathogenesis is partially attributable to OROV antagonism of innate immune signaling. We further demonstrate that OROV is vertically transmitted and infects the fetal tissues in a murine model of congenital infection. Our results demonstrate that OROV can be vertically transmitted and has heightened capacity for neurodevelopmental disruption. These findings underscore need for monitoring OROV as a re-emerging virus capable of inducing microcephaly in infected fetuses.

## INTRODUCTION

Oropouche virus (OROV) is an understudied, re-emerging virus that historically causes a self-limiting febrile illness in healthy individuals^1^. Typically transmitted by the *Culicoides* biting midge, OROV has a tri-segmented RNA genome, and as such, is capable of genetic reassortment^2^. In fact, novel OROV reassortants have been increasingly associated with recently reported adverse perinatal outcomes^3^ and disease^4,5^. As of August 2025, multiple cases of OROV vertical transmission have been confirmed and associated with fetal death and congenital anomalies^3,6,7^. Observed birth defects involve the fetal central nervous system and include severe microcephaly, cerebellar hypoplasia, ventriculomegaly, and lytic neural necrosis^8^. Despite detection of OROV RNA and antigen in fetal tissue^3^, there is no causal link between OROV infection and these pathologies, highlighting a critical need to study OROV as a congenital pathogen and to determine the impact of infection on neurodevelopment.

TORCH pathogens are infectious agents that can be transmitted *in utero*. Viruses, such as Zika virus (ZIKV), have the capacity to cross the placental barrier and infect the fetal central nervous system^9^. ZIKV has a tropism for neural progenitor cells in the fetal brain^10,11^, which are critical for proper neuronal development. As multipotent stem cells, neural progenitor cells give rise to various neuronal and glial lineages in the central nervous system. Virus infection and subsequent virus-induced dysregulation of neural progenitor cells can trigger structural brain defects, and in severe cases cause microcephaly^12^. To model microcephaly in a relevant human model, multiple groups have used iPSC-derived brain organoids to assess the impact of TORCH pathogens on human neurons^11,13–17^. Importantly, forebrain organoids mimic the first-trimester fetal brain and contain neurons of varying differentiation states, such as neural progenitor cells. Despite growing concern about OROV-induced fetal microcephaly, the impact of OROV infection on developmental neuronal dysregulation has not been characterized.

To probe the mechanism of OROV-induced microcephaly, we used forebrain organoids as a complex *in vitro* human model system. We demonstrate that both the historical TR 9760 OROV strain and more recent 240023 OROV strain robustly infect neurons of varying differentiation states in forebrain organoids, including neural progenitor cells. Notably, we demonstrate that OROV infection of early-stage forebrain organoids restricted organoid growth to induce a microcephaly-like phenotype, similar to ZIKV. Yet, distinct from ZIKV, OROV had a heightened capacity for neuroinfection and developmental disruption. We found that neurons were particularly permissive to OROV neuroinfection since OROV replication was not sensitive to IFN signaling in neurons. Lastly, using *in vivo* murine modeling, we demonstrate the capacity of OROV to cross the maternal-fetal barrier and establish infection in fetal tissues. Cumulatively, these results suggest that OROV is a vertically transmitted pathogen that can induce microcephaly-like phenotype in forebrain organoids. These data align with the rising clinical associations between OROV infection and fetal microcephaly.

## RESULTS

### OROV infects SOX2+ neural progenitor cells in human forebrain organoids

**Figure 1:**
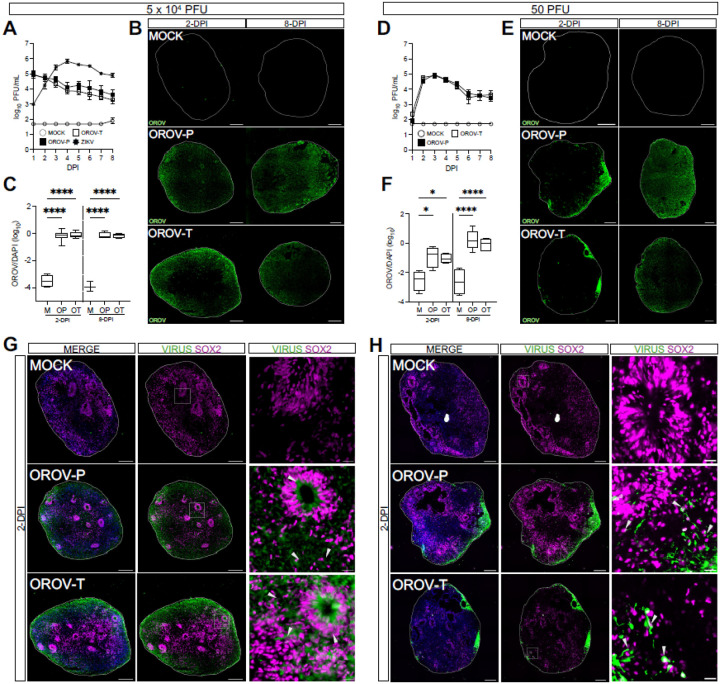
OROV infects SOX2+ neural progenitor cells in human forebrain organoids. Day-in-vitro (DIV) 35 iPSC-derived human forebrain organoids were mock-infected (MOCK or M) or infected with Zika virus (ZIKV or Z), the historical Oropouche prototypical strain (OROV-P or OP), or a recent traveler isolate of Oropouche (OROV-T or OT) at **(A-C)** 5×10^4^ or **(D-F)** 50 plaque forming units (PFU) for 24 hours. **(A, D)** Plaque assays were performed on culture supernatant through 8-days post infection (dpi) to quantify infectious virus production over time. Data presented as mean ± SEM (n ≥ 4 organoids, N ≥ 3 independent experiments). **(B, E)** Representative immunofluorescent images of organoids mock-infected or infected with OROV-P or OROV-T at 2- or 8-dpi. Organoid sections were outlined using DAPI (*blue*) and stained for OROV antigen (*green*). Scale bars = 200 μm. **(C, F)** Quantification of OROV+ organoid area relative to DAPI+ area in immunofluorescent images. Data presented as box and whisker plot; whiskers indicate minimum and maximum (n ≥ 3 organoids, N ≥ 2 independent experiments). **(G-H)** Representative immunofluorescent images of mock-, OROV-P- or OROV-T-infected for 2-dpi at **(G)** 5×10^4^ PFU or **(H)** 50 PFU. Sectioned organoids were outlined using DAPI (*blue*) and stained for OROV or ZIKV antigen (*green*) and SOX2 (*magenta*). White box indicates inset. Gray arrows indicate virus-infected SOX2+ cells. Scale bars of whole organoids = 200 μm and inset = 20 μm. (n ≥ 4 organoids, N ≥ 2 independent experiments). Statistical analysis performed with one-way ANOVA followed by Sidak’s multiple comparisons test, * p < 0.05, **p < 0.01, ***p < 0.001, ****p < 0.0001.

We first assessed whether OROV could productively infect human forebrain organoids at day-*in-vitro* 35 (35-DIV), when forebrain organoids are comprised of a mix of neural progenitor cells and more differentiated neurons (Fig. S1A). We used two strains of OROV: a historic strain, TR 9760, isolated in 1955 (OROV-Prototypical, or OROV-P), and a more recent strain, 240023, isolated in 2024 from the serum of an infected Cuban traveler (OROV-Traveler, or OROV-T)^18^. Both OROV-P and OROV-T strains exhibited robust replication in forebrain organoids and reached peak virus titers by 2-days post-infection (dpi) (Fig. 1A). In contrast, ZIKV exhibited slower viral production and reached peak titers at 4-dpi. Since we did not see an increase in OROV titers past 2-dpi, we sought to clarify if OROV can establish productive infection in forebrain organoids. Thus, we reduced virus inoculum to 50 PFU (Fig. 1D) and observed that OROV-P and OROV-T reached peak virus titers as early as 3-dpi.

Orthogonally, we analyzed OROV-infected forebrain organoids for OROV antigen at 2- and 8-dpi via immunofluorescence. At the higher inoculum concentration, we detected viral antigen at 2-dpi that persisted at 8-dpi (Fig. 1B). At the lower inoculum concentration, we detected viral antigen at the periphery of the organoid as early as 2-dpi that spread by 8-dpi (Fig. 1E, 1F), indicating the virus can efficiently disseminate and infect human forebrain organoids. However, both OROV strains shared similar infection kinetics indicating no significant differences in neuroinfection between the two strains. Collectively, these data demonstrate that OROV, regardless of strain, robustly infects and replicates in complex human forebrain organoids, underscoring its putative role in neuropathogenesis.

Forebrain organoids are comprised of multiple neurons in various stages of differentiation (Fig. S1A-D)^19^. Thus, we next sought to identify the neuronal subtypes targeted by OROV-P and OROV-T. 35-DIV forebrain organoids were infected with 5 × 10^4^ PFU (Fig. 1G) or 50 PFU (Fig. 1H) of OROV-P or OROV-T and analyzed via immunofluorescence at 2-dpi. Both OROV-P and OROV-T infected SOX2+ and SOX2− neurons, revealing capacity to infect both neural progenitor cells and differentiated neurons. Together, these data establish that OROV can productively infect human forebrain organoids with more robust replication kinetics than Zika virus. Additionally, OROV exhibits a tropism for neural progenitor cells in addition to more differentiated neuronal subtypes.

### OROV disrupts forebrain organoid development

**Figure 2:**
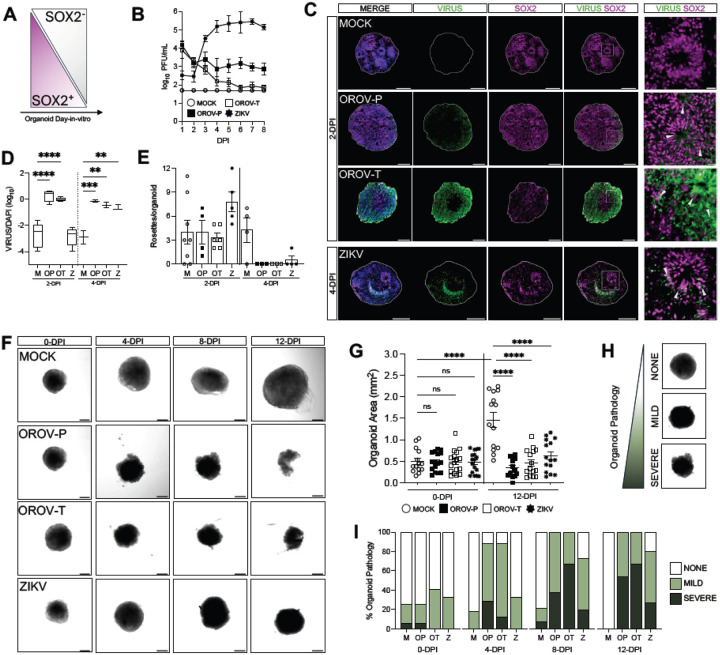
OROV disrupts forebrain organoid development. **(A)** Schematic showing the proportion of SOX2+/SOX2-cells in iPSC-derived human forebrain organoid development. **(B-I)** DIV15 forebrain organoids were mock-infected or infected with 5×10^4^ PFU of OROV-P, OROV-T, or ZIKV for 24 hours. **(B)** Viral plaque assay was performed on culture supernatant through 8-days post infection (dpi). Data presented as mean ± SEM. (n ≥ 2 organoids, N ≥ 2 independent experiments). **(C)** Representative immunofluorescent images of organoids mock-infected or infected with OROV-P, OROV-T at 2-dpi, or ZIKV at 4-dpi. Organoid sections were outlined using DAPI (*blue*) and stained for OROV or ZIKV antigen (*green*) and SOX2 neural progenitor cell marker (*magenta*). White box indicates inset. Gray arrows indicate virus-infected SOX2+ neural progenitor cells. Scale bars of whole organoids = 200 μm and inset = 20 μm. **(D)** Quantification of OROV+ organoid area relative to DAPI+ area in immunofluorescent images of organoids infected for 2- and 4-dpi. Data presented as box and whisker plot; whiskers indicate minimum and maximum (n ≥ 2 organoids, N ≥ 2 independent experiments). **(E)** Number of neural rosettes per mock- or virus-infected human forebrain organoid were quantified at 2- and 4-dpi. Data presented as means ± SEM (n ≥ 2 organoids, N ≥ 2 independent experiments). **(F)** Representative confocal images of mock or virus-infected organoids at 0-, 4-, 8-, and 12-dpi (n ≥ 13 organoids, N = 3 independent experiments). **(G)** Quantification of mock- or virus-infected organoid area at 0- and 12-dpi. (H) Organoid pathology was assessed via confocal microscopy and categorized as no, mild, or severe pathology. **(I)** Organoid pathology assessment was quantified and percentages plotted as contingency plots. Differences in categorical distribution of organoid pathology were analyzed using the Cochran-Armitage test and P values were adjusted for multiple comparisons using the Bonferroni correction (n ≥ 13 organoids, N = 3 independent experiments). All other statistical analysis were performed with one-way ANOVA followed by Sidak’s multiple comparisons test, ns p ≥ 0.05, * p < 0.05, ***p < 0.001, ****p < 0.0001.

As human forebrain organoids develop *in vitro*, the proportion of neural progenitor cells decrease as differentiated neurons are generated (Fig. 2A). As such, early-stage organoids are comprised of a greater proportion of SOX2+ neural progenitor cells in organized structures called neural rosettes^19^. Previous studies show ZIKV infection of early-stage organoids markedly impacts organoid structure^11,15,16^. Thus, to further assess impact of OROV on forebrain organoid structure and growth, we examined how infection influences organoid growth and development at 15-DIV.

15-DIV organoids were either mock-infected or infected with 5 × 10^4^ PFU of OROV-P, OROV-T or ZIKV and infectious virus production was assessed over time. Similar to 35-DIV organoids, both strain of OROV and ZIKV infect and replicate in 15-DIV forebrain organoids (Fig, 2B). OROV antigen was detected as early as 2-dpi while ZIKV antigen was detected at 4-dpi (Fig. 2C, 2D). We then assessed if virus infection impacted neural rosette formation in organoids. At 2-dpi, virus infections did not impact neural rosette counts in the organoids. However, by 4-dpi, all virus infections significantly reduced the number of neural rosettes (Fig. 2E).

To determine if neural rosette disruption impacted organoid growth, we next evaluated organoid morphology and size over 12 days of infection (Fig. 2F). While mock-infected organoids significantly grew over time (Fig. 2F, 2G), OROV- and ZIKV-infected organoids did not increase in size, with significant growth inhibition at 12-dpi (Fig. 2G). To understand whether OROV caused overt pathology in forebrain organoids, we systematically evaluated organoid pathology over time using three observable categories: ‘none, ‘mild’ and ‘severe’ (Fig. 2H) as determined by organoid integrity, circular structure, and density. At 0-dpi, all organoids displayed similar proportions of pathology, consistent with the mechanical stress of removing forebrain organoids from Matrigel. Both OROV-P and OROV-T significantly induced severe pathology in a greater number of organoids exhibiting as early as 4-dpi compared to mock- and ZIKV-infected organoids (Fig. 2I). This observation held until 12-dpi; a greater proportion of OROV and ZIKV– infected organoids exhibited severe pathology compared to mock-infected organoids. Further, more than half of OROV-infected organoids, regardless of virus strain, exhibited severe pathology, while ZIKV infection induced severe pathology in only 27% of organoids (Fig. 2I). These data indicate both strains of OROV induce neurodevelopmental disruption, organoid growth restriction, and neuropathology more rapidly and severely than ZIKV.

We hypothesized that organoid disruption may be driven by virus-induced cell death. Therefore, we next assessed if virus infection induced cell death in 15- and 35-DIV forebrain organoids by deoxynucleotidyl transferase dUTP nick end labeling (TUNEL) immunofluorescence, (Fig. S2A, S2C). We show that at 4-dpi, both OROV-P and OROV-T infection induces an increase in TUNEL staining. In contrast, TUNEL+ cells remain unchanged with ZIKV infection at 4-dpi (Fig. S2C). In 35-DIV organoids, all viruses induced cell death by 8-dpi (Fig. S2B, S2D). Collectively, these data suggest that virus-induced neuronal death likely contributes to the severity of organoid pathology observed in Fig. 2I.

### OROV antagonizes type I interferon signaling

**Figure 3:**
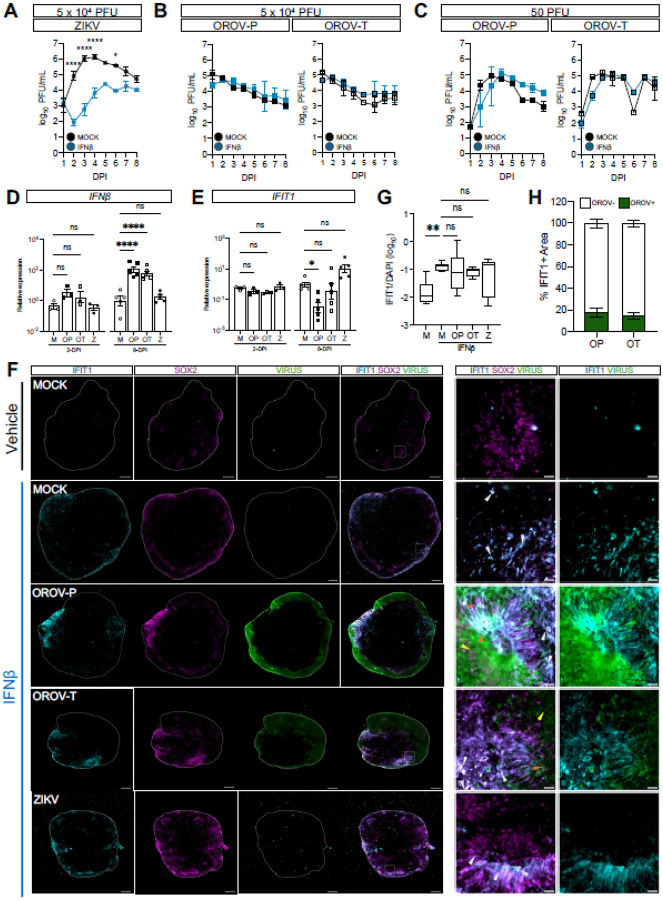
OROV antagonizes type I interferon signaling. **(A-C)** DIV35 forebrain organoids were pretreated with IFNβ or vehicle prior to infection with **(A)** 5×10^4^ PFU of ZIKV **(B)** 5×10^4^ PFU or **(C)** 50 PFU of either OROV-P or OROV-T for 24 hours. Plaque assays were performed on culture supernatant through 8-dpi. Additional IFNβ or vehicle treatments were given at 2-, 4-, and 6-dpi. Data presented as mean ± SEM. (n ≥ 2 organoids, N ≥ 2 independent experiments). **(D-E)** DIV35 organoids were mock-infected (M) or infected with 5×10^4^ PFU of OROV-P (OP), OROV-T (OT), or ZIKV (Z). Organoid RNA was harvested at 2- and 8-dpi and RT-qPCR was performed for **(D)** *IFNβ* and **(E)** *IFIT1*. **(F)** Representative immunofluorescent images of DIV35 forebrain organoids mock-infected, or infected with 5×10^4^ PFU of OROV-P, OROV-T or ZIKV for 24-hours prior to stimulation with IFNβ or vehicle for 24-hours. Organoid sections were outlined using DAPI *(blue)* and stained for IFIT1 protein (*gray*), SOX2 neural progenitor cell marker (*magenta*), and OROV or ZIKV antigen (*green*). White box indicates inset. Orange arrows indicate virus-infected IFIT1+ cells. Yellow arrows indicate virus-infected IFIT- cells. Gray arrows indicate IFIT+ uninfected cells. Scale bars of whole organoids = 200 μm and inset = 20 μm. **(G)** Quantification of IFIT1+ area relative to DAPI+ area in immunofluorescent images of organoids in **(F)**. Data presented as box and whisker plot; whiskers indicate minimum and maximum. (n ≥ 7 organoids, N = 2 independent experiments). **(H)** Quantification of IFIT1+ area that was either OROV negative or OROV positive of organoids in **(F)**. Statistical analysis for **(A-C)** were performed with ordinary two-way ANOVA followed by Sidak’s multiple comparisons test. All other analyses were performed with one-way ANOVA followed by Sidak’s multiple comparisons test, ns p > 0.05, * p < 0.05, **p < 0.01, ****p < 0.0001.

We next sought to explore why forebrain organoids were particularly permissive to OROV-P and OROV-T neuroinfection. We have previously identified neural progenitor cells as important bystander cells that prevent viral replication and spread^20^. Given the intrinsic antiviral capacity and pronounced OROV tropism for neural progenitors, we investigated whether exogenous induction of type I IFN signaling could suppress OROV infection. Specifically, we pretreated organoids with a representative type I IFN, IFNβ, 6-hours prior to mock-infection or infection with 5×10^4^ PFU of ZIKV, OROV-P or OROV-T. We then stimulated with IFNβ every other day while harvesting organoid supernatant daily until 8-dpi. We found that IFNβ treatment significantly inhibited ZIKV virus production at multiple timepoints, indicating that ISGs induced by IFNβ restrict ZIKV replication in forebrain organoids (Fig. 3A). However, IFNβ treatment did not impact OROV-P or OROV-T virus production at any of the timepoints studied (Fig. 3B). This finding was consistent when organoids were infected with 50 PFU of OROV (Fig. 3C). Collectively, our findings reveal that OROV infection is not limited by type I IFN signaling in organoids.

Similar to other bunyaviruses, OROV has a nonstructural protein that antagonizes type I IFN signaling in host cells^21^. We therefore next sought to understand if OROV infection antagonizes ISG production in forebrain organoids. We mock-infected or infected 35-DIV forebrain organoids with 5×10^4^ PFU of OROV-P, OROV-T or ZIKV for 2- or 8-days. We found that infection with either OROV-P or OROV-T did not impact *IFNβ* transcripts at 2-dpi, but significantly increased *IFNβ* transcripts at 8-dpi (Fig. 3D). This was in contrast with ZIKV-infected organoids, which did not increase *IFNβ* transcripts at either time point. We next analyzed the impact of OROV infection on the downstream ISG, IFN-induced protein with tetratricopeptide repeats 1 (*IFIT1*) to determine if upregulation of *IFNβ* impact downstream ISG induction. Notably, we show that only OROV-P infection downregulates *IFIT1* expression at 8-dpi (Fig. 3E).

To directly investigate OROV-induced type I IFN antagonism in neurons, we next assessed the ability of OROV infected neurons to induce IFN signaling. To do this, we mock-infected or infected forebrain organoids with OROV-P, OROV-T or ZIKV at 5×10^4^ PFU for 24-hours followed by stimulation with exogenous IFNβ for 24-hours. We then determined the expression of IFIT1 protein in actively infected neurons versus bystander non-infected neurons via immunofluorescence. If OROV actively antagonizes IFN signaling, we would expect restricted IFIT1 protein expression in infected cells, while uninfected bystander cells would retain IFIT1 expression. Exogenous IFNβ induced IFIT1 protein expression in SOX2+ neural progenitor cells of mock-infected and infected organoids (Fig. 3F, 3G). But importantly, OROV-infected neural progenitor cells largely did not express IFIT1 24-hours after IFNβ-stimulation (Fig. 3F, 3G). In fact, approximately 82% of the IFIT1+ regions of infected organoids were OROV- (Fig. 3H).

However, bystander non-infected neural progenitor cells expressed the IFIT1 protein, suggesting active OROV infection limits IFIT1 protein induction. Given the short period of infection, ZIKV antigen was mostly undetected, limiting the comparison of infected versus bystander cells. Altogether, these data suggest OROV neuroinfection is particularly permissible as OROV replication is not sensitive to the repertoire of ISGs induced by neurons and actively antagonizes type I IFN signaling in infected neurons.

### OROV is vertically transmitted to the developing fetus

**Figure 4:**
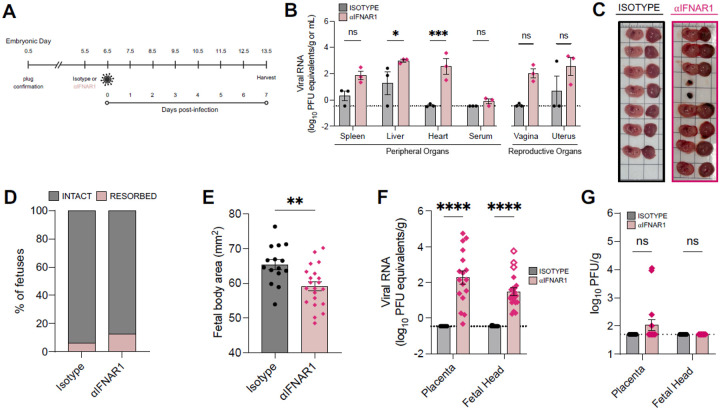
OROV is vertically transmitted to the developing fetus. **(A)** Schematic of experimental design. 9–12-week-old female pregnant C57BL/6 mice (dams) were treated with either 0.5mg/mouse of αIFNAR1 (MAR1–5A3) or isotype control (mouse IgG1) intraperitoneally on embryonic day 5.5 (E5.5). On E6.5, dams were infected with 10^4^ PFU of OROV-P (strain TR9760) subcutaneously via footpad injection. **(B-G)** All maternal and fetal tissues were analyzed at 7dpi (E13.5). **(B)** Peripheral maternal tissue (spleen, liver, heart, serum) along with female reproductive tissue (vagina, uterus) were harvested and OROV RNA in peripheral and reproductive maternal tissues quantified via RT-qPCR (n=3 dams per condition, N=3 independent experiments). **(C)** Representative images of matched fetuses and placentas from isotype-and αIFNAR1-treated dams. **(D)** Quantification of resorbed and intact fetal tissue **(E)** Quantification of fetal body area. **(F)** OROV RNA in placenta and fetal heads were quantified via RT-qPCR. Open symbols denote fetal resorptions. **(G)** Viral titers in placenta and fetal heads were quantified via plaque assay. Dashed line indicates limit of detection for each assay (n>14 samples per condition, N=3 independent experiments). Statistical analysis for **(F)** was performed with an unpaired t-test. All other analyses were performed with two-way ANOVA adjusted for multiple comparisons, ns p > 0.05, * p < 0.05, ***p < 0.001, ****p < 0.0001.

The clinical manifestations of OROV infection in fetuses from infected pregnant women, coupled with our work establishing the ability of OROV to induce neurodevelopment disruption in forebrain organoids led us to explore the potential of OROV vertical transmission and fetal infection *in vivo*. We, and others, did not detect robust OROV dissemination in pregnant C57BL/6 (WT) dams^22^. Therefore, to promote viral dissemination in a pregnant dam, we transiently depleted type I IFN signaling using an IFNAR1-blocking antibody (αIFNAR1) one day prior to infection to acutely permit productive OROV replication and viral dissemination. Specifically, we treated 9–12-week-old pregnant females with 0.5 mg αIFNAR1 or isotype-control antibody 24-hours prior to a footpad infection of 10^4^ PFU/mouse of OROV-P at embryonic day 6.5 (E6.5). We then monitored mice for 7 days (Fig. 4A) and harvested maternal peripheral tissue (spleen, liver, heart, serum), maternal reproductive tissue (vagina, uterus), and fetal tissue (placenta, fetal heads) on E13.5. Notably, we detected increased OROV RNA in all maternal peripheral and reproductive tissues (Fig. 4B) of αIFNAR1-treated compared to isotype-treated C57BL/6 dams. Interestingly, we noted a difference in fetal pathology in αIFNAR1-treated compared to isotype-treated dams (Fig. 4C), where we observed a slight increase in fetal resorption rate (Fig. 4D) and a significant reduction in fetal body area (Fig. 4E). We detected OROV RNA in the placenta and fetal heads of αIFNAR1-treated dams, this is in stark contrast with isotype control (Fig. 4F). Notably, we found resorbed fetuses (demarked by open diamonds) to have higher viral loads than all intact fetal heads (Fig. 4F). Lastly, we detected infectious virus by plaque assay in a subset of placental samples from αIFNAR1-treated dams, whereas no placentas from control dams contained infectious OROV (Fig. 4G). Notably, the placentas with infectious OROV detectable by plaque assay contained the greatest amount of OROV RNA by RT-qPCR (Figure S3A), suggesting that the amount of OROV RNA found in fetal tissues at this infection timepoint may not have yet reached a threshold for infectious virus to be detected via plaque assay. Altogether, our data indicate that transient blockade of type I IFN signaling permits vertical transmission of OROV resulting in fetal tissue infection and fetal growth restriction.

## DISCUSSION

Historically, human OROV infection has led to self-limiting febrile illness^1^. However, the 2022–2024 OROV outbreak has been increasingly associated with adverse neurotropic complications in adults^4,5^, vertical transmission, and fetal abnormalities^8^. These growing clinical associations signal the possible emergence of a new TORCH pathogen. We designed this study to investigate OROV infection-induced neurodevelopmental disruption and putative congenital infection capacity. We demonstrate that OROV robustly infects neural progenitor cells and more differentiated neurons in iPSC-derived forebrain organoids, a human model of early fetal brain development. We find that OROV infection in early-stage forebrain organoids induces severe pathology and microcephaly-like phenotype, as defined by reduced organoid size and ablation of neural rosette formation. Notably, OROV had a heightened capacity for neuroinfection, and developmental disruption as compared to the neurotropic, TORCH pathogen, ZIKV. OROV infection also antagonized type I IFN signaling early in neuroinfection which may contribute to this increased pathogenesis. Lastly, by establishing a new *in vivo* model of robust congenital infection, we show that OROV can cross the maternal-fetal barrier to establish infection in both the placenta and fetus. Further, our *in vivo* model associates murine OROV congenital infection with fetal growth restriction.

The recent OROV outbreak is speculated to be caused by a novel viral reassortment event leading to increased clinical cases^7^. Previous work using an *Ifnar1*^*−/−*^ mouse model^22^ found novel strains of OROV to be more permissive and virulent. Yet, our data suggest the historic OROV-P and novel OROV-T infection of forebrain organoids do not exhibit strong differences in virus replication and virus-induced pathology. These conflicting data could be due to differences of OROV replication dynamics in mouse versus human model systems, or distinctions in the host immune capacity of infection model. Further work is needed to assess how strain differences are linked to increased incidence of OROV fetal neuroinfection. Interestingly, OROV-T was found to better replicate in midges, the primary OROV vector, as compared to a historic OROV strain^23^. It is also therefore plausible that novel OROV strains may cause increased human disease indirectly through increased vector infectivity.

Regardless of strain, we observed that OROV infection is heightened compared to ZIKV infection in forebrain organoids. We show both OROV strains efficiently infect neural progenitor cells and other neurons in the forebrain organoid. In contrast, ZIKV infection was largely restricted to neural progenitor cells and exhibited slower viral replication kinetics. We also show that type I IFN signaling limits ZIKV infection, but not OROV infection in the forebrain organoid model. Enhanced antagonism of type I IFN signaling by OROV may explain its widespread neurotropism compared to ZIKV. Additionally, OROV entry into neurons exploits the entry factor Lrp1^24^, a mechanism absent in ZIKV infection. This may explain the expanded tropism of OROV for both SOX2+ and SOX2− cells in the organoid model. This difference in neurotropism, early infection immune antagonism, and differences in viral entry may explain why OROV infection leads to increased neuron death and organoid pathology.

Additionally, we developed a congenital murine model of OROV infection. In immunocompetent mice, we show that type I IFN signaling is important in controlling viral dissemination, since transient depletion of type I IFN signaling in pregnant dams allowed for vertical transmission and fetal infection. These data indicate that in mice, IFNAR signaling is important for virus dissemination to fetal tissues. Of note, OROV neuroinfection in our organoid model was not impacted by active type I IFN signaling. This suggests that type I IFN signaling is important for neuroinvasion, but not infection. This is likely due to the differential antiviral capacities, or repertoire of ISGs induced by different cell types (i.e. monocytes versus neurons). Alternatively, this could be due to species differences in antiviral dynamics between mouse and human, which is known to occur with ZIKV infection^25,26^. However, further experiments are needed to explore this difference.

Cumulatively, our work is the first to define OROV as a TORCH pathogen capable of neurodevelopmental disruption by targeting neural progenitor cells and suppressing innate antiviral responses. We further show that a novel strain reassortment did not impact direct neuroinfection. These data align with the rising clinical associations between OROV infection and fetal microcephaly. The work presented here underscores the significance of monitoring OROV as a re-emerging virus capable of *in utero* transmission.

## METHODS

### Viruses and cells

OROV-P, TR-9760, was purchased from ATCC (VR-266), while OROV-T, strain 240023, was obtained from BEI resources, NIAID, NIH, (NR-59930). OROV stocks were propagated in Vero cells. African green monkey kidney (Vero) (ATCC, Cat#CCL-81) cells were grown and maintained in Dulbecco’s modification of eagle’s medium (Corning, Cat#10–013-CV) supplemented with 10% fetal bovine serum (Sigma-Aldrich, Cat#F2442) and 1% penicillin–streptomycin (ThermoFisher Scientific, Cat#15140122). ZIKV Cambodian FSS13025 strain (ZIKV-CAM) was obtained from the World Reference Center for Emerging Viruses and Arboviruses at University of Texas Medical Branch, Galveston. ZIKV-CAM stocks were propagated in C6/36 mosquito (*Aedes albopictus*) cells. C6/36 cells (ATCC, CRL-1660) were maintained in Dulbecco’s Modified Eagle’s Medium (ThermoFisher, 11965092) supplemented with 10% heat-inactivated fetal bovine serum (FBS) (ThermoFisher, A5670701), 1% penicillin/streptomycin (ThermoFisher, 15140122) and 1% tryptose phosphate broth (Sigma-Aldrich, T8159) at 30°C. For high-titer doses, ZIKV and OROV were concentrated by ultrafiltration (Centricon Plus-70; MWCO: 30,000). Human-induced pluripotent stem cell line C1–2^27^ was maintained in mTSeR media (StemCell Technologies, Cat#100–1130) prior to forebrain organoid generation. Cell lines were authenticated by morphology and were routinely tested for mycoplasma contamination.

### Mice

C57BL/6 (wild-type; WT) mice were purchased from The Jackson Laboratory (strain #000664) and maintained at the University of Pennsylvania under specific pathogen-free conditions. Timed matings were performed by mating >8-week old female WT mice to male WT mice overnight. Subsequently male and female mice were separated the following morning after checking for the presence or absence of a copulation plug, with plug observation date designated E0.5. Female mice with a copulation plug were used for congenital OROV infection experiments. All experiments were performed following IACUC guidelines.

### *In vivo* OROV Infections

9–12-week old pregnant female C57BL/6 WT mice were treated intraperitoneally with 0.5mg/mouse of either an αIFNAR1-blocking antibody (clone MAR1–5A3; BioXCell Cat#BE0241) or an IgG1 isotype-control antibody (BioXCell Cat#BE0083) on E5.5. αIFNAR1-pregnant female WT mice were then infected subcutaneously via footpad with 10^4^ PFU of OROV-P (strain TR9760) on E6.5. Mice were monitored daily for weight changes and sacrificed 7 dpi, on E13.5. Tissues were isolated, placenta and fetuses were photographed on measurement paper using an iPhone camera, and all tissues were collected into tubes containing 1mL of DMEM 2%FBS, 1% penicillin/streptomycin, 1% HEPES + silica beads, homogenized, and analyzed for subsequently for viral loads.

### Generation of forebrain organoids

Forebrain organoids were generated using a previously described protocol^19,20^. In short, iPSCs were maintained and incubated at 5% CO2, 37 °C. iPSCs exhibiting signs of differentiation were excluded from organoid generation. At 0-DIV, iPSCs were seeded in low-attachment 96-well plates (Corning, Cat#3474) at a density of 100,000 cells/well in mTeSR1 supplemented with 10 μM of ROCK inhibitor (StemCell Technologies, Cat#72304). At 2-DIV, embryoid bodies (EB) were transferred using cut 200uL pipette tips into 6-well plates in DMEM:F12 media (Gibco, Cat#11320033), supplemented with 20% Knockout Serum Replacement (Gibco, Cat#10828028), 1X GlutaMAX, 1X MEM Non-essential Amino Acids (Gibco, Cat#11140–050), 1X EmbryoMax 2-Mercaptoethanol (Sigma-Aldrich, Cat#ES-007-E), 1X Penicillin/Streptomycin, 1uM LDN193189 (StemCell Technologies, Cat#72147), 1 mM SB-431542 (StemCell Technologies, Cat#72234) and 2 μg/mL 0.2% heparin solution (StemCell Technologies, Cat#7980). Media changes were performed daily. At 6-DIV, EB media was replaced with induction media consisting of DMEM:F12, 1X N-2 Supplement (ThermoFisher Scientific, Cat#17502048), 1X Penicillin/Streptomycin, 1X NEAA, 1X GlutaMAX, 1 μM CHIR99021 (StemCell Technologies, Cat#72054), and 1 μM SB-431542. Round EBs with bright edges were selected at 7-DIV, coated with Matrigel, and plated on ultra-low-attachment 6-well plates (Corning, Cat#3471). Media was changed every other day until 14-DIV when EB-Matrigel complexes were disassociated. EBs, now organoids, were maintained until either 15-DIV or 35-DIV with daily media changes consisting of 1:1 Neurobasal media and DMEM/F12 supplemented with 1X N-2, 1X B-27, 1X GlutaMAX, 1X MEM Non-essential Amino Acids, 1X EmbryoMax 2-Mercaptoethanol, 1X Penicillin/Streptomycin and 2.5 μg/mL of insulin (Sigma-Aldrich, Cat#I9278). All EBs and organoids were maintained at 5% CO2, 37 °C and incubated on a shaking platform at 120 revolutions per minute, except when EBs were complexed with Matrigel.

### *In vitro* forebrain organoid virus infections

Forebrain organoids (15-DIV or 35-DIV) were placed in 24-well tissue culture treated plates with two organoids per well. Culture media was completely removed and replaced with fresh culture media containing 5×10^4^ or 50 plaque forming units (PFU) of virus as indicated. Organoids were infected for 24-hours at 37°C with complete media changes daily until samples were harvested. For cumulative virus quantification, organoid media remained unchanged.

### Interferon treatments

35-DIV forebrain organoids were pretreated 100 IU/mL of recombinant human IFNβ (PeproTech, Cat#300–02BC) for 6-hours prior to virus infection. After 24h of infection, virus containing media was removed and replaced with fresh media. Media was then supplemented with 100 IU/mL recombinant IFNβ at 2-, 4-, and 6-days post-infection.

### RNA extraction, cDNA generation and quantitative RT-PCR

Forebrain organoids were homogenized in TRIzol ((Invitrogen, Cat#15,596,018) and RNA was subsequently extracted using Clean and Concentrator kit (Zymo, Cat#R1017) as per the manufacturer’s instructions. RNA concentration was quantified using the ThermoFisher Scientific Nanodrop One spectrophotometer. cDNA was generated using 200 ng of RNA in iScript cDNA synthesis kit (Bio-Rad, Cat#1708890) following manufacturer’s instructions. Quantitative RT-PCR was performed with Power SYBR Green Master Mix (ThermoFisher Scientific, Cat#4367659). Reactions were run via QuantStudio3 (50 °C: 2’; 95 °C: 10’; 40 × 95 °C: 15 s, 60 °C: 1’) with the addition of a final melt curve (95 °C: 15 s; 60 °C: 1’; 95 °C: 1’). All samples were loaded in technical duplicates. Average threshold cycle (Ct) value was calculated per sample for *IFNβ* ((F: 5′-CTTTGCTATTTTCAGACAAGATTCA-3′, R: 5′-GCCAGGAGGTTCTCAACAAT-3′) or *IFIT1* (F: 5′-TACAGCAACCATGAGTACAA-3′, R: 5′- TCAGGTGTTTCACATAGGC-3′), which was normalized against expression of housekeeping gene, *HPRT* (F: 5′-CATTATGCTGAGGATTTGGAAAGG-3′, R: 5′-CTTGAGCACACAGAGGGCTACA-3′), and presented as fold change.

To quantify OROV RNA in infected mouse tissues, 200μL of tissue homogenate or serum was added to 600μL of TRIzol, frozen at −80, and RNA was subsequently extracted using Phasemaker (Invitrogen; #A33251) tubes according to manufacturer’s protocol. Total RNA was then quantified as above, and cDNA was generated as above using 1μg total RNA per reaction. OROV cDNA was quantified by as above by RT-qPCR using previously published primers targeting the S segment of OROV (F: 5′- GCGTCACCATCATTCCAAGTA-3′, R: 5′-CCCAGATGCGATCACCAATTA-3′)^28^. Ct values were fitted to a standard curve generated from a known concentration (PFU/mL) of stock OROV-P to determine the PFU equivalents/mL of a given sample. Samples were then normalized to respective starting mass (g) or volume (mL) and reported as PFU equivalents/g or mL.

### Plaque assay

Vero cells were seeded at a density of 5×10^5^ cells/well in a 6-well plate overnight. After 24-hours, supernatants were harvested from infected forebrain organoids and serially diluted in DMEM (Corning, Cat#10–013-CV) supplemented with 10% heat-inactivated Fetal Bovine Serum (Sigma-Aldrich, Cat#F2442) and 1% Penicillin/Streptomycin (ThermoFisher Scientific, Cat#15140122). Seeded Vero cells were treated with 200μL of diluted supernatants and incubated for 1-hour, with rotations every 15 minutes. Following incubation, inoculum was aspirated and replaced with MEM (Sigma-Aldrich, Cat#11430030) supplemented with 5% FBS, 1% GlutaMAX, 1% Non-essential amino acids, and 0.65% agarose (Lonza, Cat#50111). OROV- and ZIKV-infected cells were fixed at 4- and 7-DPI respectively with 2 mL 10% NBF (ThermoFisher Scientific, Cat#22050105) and visualized using 0.1% crystal violet (ThermoFisher Scientific, Cat#C581–25). Plaques were manually counted, and virus titer was calculated.

### Immunofluorescence

Human forebrain organoids were incubated in 4% Paraformaldehyde solution (Thermofisher, Cat# J19943.K2) for 30 min at room temperature, while rocking, followed by overnight incubation in 30% sucrose. Fixed organoids were embedded in OCT (Sakura, Cat#4583) and cryosectioned (10 μm) using a Leica Cryostat. Slides were washed three times with PBS prior to membrane disruption with PBST (PBS supplemented with 0.4% Triton X (Sigma-Aldrich, Cat#T8787)). Sample was blocked with PBS supplemented with 1% bovine serum albumin (Sigma-Aldrich, Cat#B2518) and 2% normal donkey serum (Jackson, Cat#017-000-121) for 1-hour prior to overnight incubation with a combination of 1:200 mouse anti-OROV immune ascitic fluid (ATCC, Cat#VR-1228AF) 1:100 mouse Anti-Flavivirus Group Antigen Antibody (Millipore, Cat#MAB10216-I-25UG), or 1:100 rabbit anti-IFIT1 (CST, Cat#14769) diluted in blocking buffer. Samples were washed three times for 10 min with PBST. After washing, samples were incubated for 2-hours in a combination of 1:1000 goat anti-mouse Cy7 (Thermofisher, Cat#A-21037), 1:1000 goat anti-rabbit Cy7 (Thermofisher, Cat#A21039), 1:1000 rabbit monoclonal anti-SOX2 (CST, Cat#5067) and 1xDAPI (ThermoFisher Scientific, Cat#AC202710100) diluted in PBST. Following incubation, sections were washed with PBST and ultrapure water. Sections covered with mounting media and a coverslip was applied and sealed. Images of organoids were acquired using a Nikon Ti2E scope. Image processing was conducted using ImageJ software.

### Organoid Size Quantification, Pathology Scoring, and Rosette Counting

Organoids were imaged using a 10X or 4X brightfield microscope depending on organoid size. All organoids were outlined and organoid area measured using ImageJ. Using a single-blinded process, healthy organoids with no pathology were characterized by clearly defined edges with no blebbing or hypodensities. Organoids with mild pathology were classified by the presence of blebbing, a lack of hypodense areas, and generally rounded organoid structure. Organoids with severe pathology were categorized by the presence of blebbing, marked hypodense areas, disruption of round structure and organoid disintegration. Lastly, organoid neural rosettes as demarcated by well-organized SOX2+ neural progenitor cells were quantified in a separate single-blinded study.

### Fetal size quantification

Fetus size from αIFNAR1-treated and isotype-control-treated WT dams were quantified using photos of respective litters and converting pixels to millimeters (mm). Fetal crown-rump length, the direct length from the edge of the fetal head to the start of the tail, was multiplied by occipital diameter, the direct length from the front to the back of the fetal head, to quantify overall fetal size. All analysis was performed using ImageJ software.

### Data analysis

All graphs were plotted, and statistical analyses were performed using GraphPad Prism. Data are expressed as Mean ± error of the mean (SEM) or as box and whisker plot; whiskers indicate minimum and maximum. Number of biological samples used per experiment (n), number of individual experiments (N), and statistical tests used for each experiment are included in figure legends. Statistical significance was determined by Unpaired Student’s t tests for group means, One-Way or Two-Way ANOVA followed by post-hoc multiple comparisons test as indicated. Differences in categorical distribution of organoid pathology were analyzed using the Cochran-Armitage test and P values were adjusted for multiple comparisons using the Bonferroni correction. p < 0.05 was considered as significant. ns = non-significant, *p < 0.05, **p < 0.01, ***p < 0.001, ****p < 0.0001.

## Supplementary Files

This is a list of supplementary files associated with this preprint. Click to download.

- BannermanOROVSInfo.pdf
